# Enhancing second-order empathy in medical practice by supplementing patients’ narratives with certainties

**DOI:** 10.1186/s12909-018-1145-y

**Published:** 2018-03-14

**Authors:** José María Ariso

**Affiliations:** Universidad Internacional de La Rioja, Almansa 101, 28040 Madrid, Spain

**Keywords:** Empathy, Certainty, Narrative, Patient care, Medical education

## Abstract

Most scholars agree that empathy is one of the keys for medical education, but it is not yet clear precisely how this term should be defined. Currently, the predominant tendency in this area consists in considering empathy within the context of narrative medicine or, more specifically, within the interaction theory instead of the simulation theory of empathy. A significant development of the interaction theory is “second-order empathy”. After describing the outlines of this kind of empathy, I suggest that the practitioner should also inquire about the patient’s certainties – in Wittgenstein’s sense – in order the better to enrich and understand her narrative. Besides offering examples of how certainties may contribute to reaching a clearer perspective of the patient’s narratives and, thus, to strengthen second-order empathy with her, guidelines are provided to train medical students in identifying such certainties.

## Background

Most medical educators are familiar with the strong decline in idealism and altruistic aspirations shown by many students during training. This change in attitude is ironically reflected in the old saying according to which medical education is divided into a “precynical” and a “cynical” phase [1]. Thus, 75% of medical students become more cynical regarding not only academic life, but also the medical profession, throughout their progress in medical school [2]. Students recognized that cynicism constitutes a coping or survival strategy to acquire greater control of their emotions and the situation itself, and by extension, to avoid professional burnout [3–5]. It could be expected that this increase of cynicism during medical training necessarily goes together with a decrease of empathy, but this is not always the case, as some medical students maintained empathy scores throughout their training [6–8]. Conversely, it has also been shown that empathy continues to decline during medical training [9, 10]: in this vein, studies worldwide have indicated that empathy is lower in men and among students who do not opt for a people-oriented specialty [11–13]. This decline in empathy is a serious problem, for regardless of how excellent technical assistance or interventions may become, quality healthcare will not be provided if physicians lack empathy [14]. Indeed, empathy not only improves the therapeutic results as well as the patient’s quality of life, [15–17] but it also provides more diagnostic accuracy and an increased sense of well-being to the physician [18–20]. It has been argued that the aforementioned drop in empathy is due to the attitude that medical students are expected to develop, above all around the third year of medical training. Specifically, students must assimilate a vast amount of information; they think that emotions might distract them from taking appropriate decisions, are constantly exposed to tragedy, fear failure, and lack role models who show how relevant empathy should be in medical practice [3, 21–24]. Yet it should not be forgotten that, in order to promote a patient centered approach, students are also encouraged not to display their own emotions.

Taking into account the importance of promoting empathy in the healthcare curriculum, a number of strategies have been used to enable practitioners to strengthen advanced communication skills with patients, e.g. in cancer care [25–27]. Literature on educational interventions aimed at enhancing empathy in undergraduate medical education reveals that such interventions may be effective in enhancing and maintaining empathy [28]. Most of these interventions dealt with patients’ narratives, but this article is intended to show how the educational interventions can be supplemented by bringing up Wittgenstein’s notion of “certainty” [29]. To begin with, I will explain why some authors have rejected the simulationist approach to train medical students in enhancing empathy and have instead opted for an interactive and narrative-based approach. Next, it will be shown that Stanghellini is fully in line with this interactive approach when he presents the term “second-order empathy” for the purpose of developing a narrative shared by the patient and doctor [30]. Subsequently, I will expound the basic lines of Wittgenstein’s notion of “certainty”, with particular attention to its similarities with and differences from the term “narrative”. On this basis, I will refer to specific cases in order to clarify how both concepts can contribute to fostering second-order empathy between the patient and the practitioner. Lastly, I will offer some guidelines to teach medical students how to empathize by taking into account their patients’ certainties, so that students learn to identify such certainties and know the extent to which they can facilitate, when appropriate, the variation of some certainties.

## From interaction theory to second-order empathy

The traditional dichotomy between natural sciences and human sciences can also be found within medical education. Since humanities play a role that is subordinate to the biomedical side, it should not be surprising that empathy is often regarded as a peripheral issue and not as a relevant aspect of the physician’s education [31–33]. Practitioners have embodied a “scientific attitude” which has been emulated by students. This attitude focuses on detachment and scientific objectivism, to the extent that empathy towards the patient should be avoided. Even though such an attitude has undoubtedly improved scientific and technological progress, it becomes especially problematic when it is applied to medical practice [34]. Fortunately, this attitude is increasingly considered as an outdated view of current communication skills training. Anyway, in order to overcome the scientific attitude in medical practice, empathy must be part of the doctor’s mindset. Keeping this in mind, the question is to discern what kind of empathy is the most suitable for overcoming the scientific attitude. In this regard, it should be noted that diverse simulation theories – henceforth “ST” – of empathy have emerged over the last years, [35–37] according to which the mental states of other people are understood by simulating them. Roughly speaking, we try to acquire other people’s perspective by simulating their actions within ourselves, and then projecting the resulting mental states into them. However, we seem to merely reiterate ourselves when the observation of what other people do or feel becomes transformed into an inner representation of what we would do or feel in such a situation [38]. This means that physicians can understand patients only inasmuch as they are successful in mimicking their patients’ mental states, i.e. the patient’s experiences are thus understood not from his very perspective, but from the practitioner’s point of view [34]. As a result of this, one could attribute too much of her knowledge, experiences and skills to the other [39]: for instance, it could be then presupposed that the patient possesses a vast amount of medical knowledge although he usually ignores which symptoms are relevant and which treatments are possible [34]. Worse still, ST promotes a scientific attitude towards understanding the patient, for he is still considered as an object that is observed and judged instead of being regarded as an individual with whom physicians should interact [34].

If empathy consists in understanding others, ST does not seem to contribute to achieve such understanding. ST should help to know the interlocutor’s mental state, yet ST is feasible only if we know in advance which mental state should be simulated. Hence, narrative competency constitutes a more suitable account of empathy than ST, [40] because this competency provides a “massive hermeneutical background”, [41, 42] i.e. learned skills and practical knowledge regarding what to expect from other people and, by extension, how to interact with them. It is therefore no surprise that current communication skills training is aimed at fostering narrative competence rather than teaching medical students to simulate patients’ mental states. Gallagher’s interaction theory – hereafter “IT” – has been regarded as the best alternative to ST, for narratives are progressively refined through our interactions with others in such a way that they can be applied to understand patients from very different backgrounds in a wide variety of situations [34]. In this way, physicians understand the patient’s mental state by situating her in the narratives acquired throughout their lives, so that they will already know what to expect from that patient given her actions and situation. The problem is that the scope of narrative competence depends on our narratives and experiences, so that, as could be expected, it is not a panacea but a limited resource that cannot be effective in all cases, particularly when the patient and the practitioner inhabit very different life-worlds. At the end of this section I will come back to this problem by indicating the direction in which an interesting solution may be found.

In my opinion, it can also be argued that ST constitutes an account of sympathy, whereas IT is closely related to empathy. Sympathy requires experiencing the patient’s emotions, but empathy consists merely in imagining what it would be like to feel such emotions [43–45]. While sympathizing practitioners must share the patient’s suffering, which may cause lack of objectivity and a huge emotional fatigue, the naturally empathic doctor is able directly to perceive the patient’s intentions and emotions in his embodied behavior, [46] thereby achieving a faster and stronger therapeutic effect [15].

An even more important advantage of IT is that ST requires the practitioner to separate herself from the patient in order to see him as a scientific object of study; conversely, IT consists in understanding the patient by interacting with him, which makes it possible for the patient to tell his story [47]. It is on this basis that physician and patient communicate with each other and gradually develop a common narrative [48]. Thus, clinical interviews should not simply be aimed at gathering data and details about a given case, but also at entering the patient’s life-world by being sensitive to different interpretations of symptoms and patient stories [49]. This dichotomy is also evident in psychiatric interviews, for there is a clearly discernible distinction between structured or symptom-oriented styles and unstructured or insight-oriented interview approaches [50]. The currently prevailing style of psychiatric interview fosters “de-narratization”, as structured interviews search for signs and symptoms that make nosographical diagnosis possible, hardly paying attention to the personal problems that arise in the rapport [30]. Taking as a reference the above-mentioned scientific attitude, these interviews are usually intended to assess bits of behavior and expression, thereby neglecting personal narratives. Whereas this third-person approach to psychiatric interviewing emphasizes objectivity and quantification and attempts to explain symptoms by reducing them to causes, the first-person approach is aimed at empathically understanding the patient’s experience. At this point, Stanghellini distinguishes between two kinds of empathy [30]. On the one hand, he refers to the *non-conative empathy* that takes place spontaneously without requiring any voluntary effort due to the resonance between two embodied selves. On the other hand, and keeping in mind that we often cannot make sense of other people’s behavior, it is then necessary to develop *conative empathy* by making the physician’s personal knowledge and past experiences resonate with those of the patient. But non-conative empathy turns out to be too naïve to understand psychotic experiences, whereas conative empathy entails the risk that the practitioner projects his own experiences onto the patient. To overcome these shortcomings, Stanghellini proposes as an alternative what he calls “second-order empathy”, which requires to begin by acknowledging that the life-world inhabited by the patient is very different from one’s own [30]. In fact, Stanghellini emphasizes the necessity of admitting “the ontological difference” between the patient’s way of being in the world and the physician’s one, as the patient lives “in a life-world whose structure is (at least in part) different” from the doctor’s life-world [30]. Although second-order empathy has its origin in psychiatric interviewing, it may be regarded as the beginning of a solution to the above-mentioned problem of the limited scope of narrative competence by drawing attention to the ontological dimension. To delve further into second-order empathy, in the next section I will explain how the use of the terms “narrative” and “life-world” can be supplemented with Wittgenstein’s notions of “certainty” and “world-picture”, which may in turn help enhance second-order empathy precisely because they reveal particularly clearly the ontological differences between the patient’s and the practitioner’s ways of being in the world as well as between their views of it.

## Narratives and certainties: A comparison and analysis of their contribution to second-order empathy

All of us need to find sense and meaning in what we do, experience and believe, for which we rely mainly on storytelling. That is why narratives “establish a form of organization in autobiographical memory providing temporal and goal structure, combining personal experiences into a coherent story related to the self” [30]. Narratives become refined and enriched over time, thus allowing us to face increasingly complex situations. It is therefore impossible to interact with other people unless we all make sense of the world through joint-narratives that ultimately enable us to understand the reasons underlying other people’s actions and intentions as well as ours. We can actually understand people who are very different from us because their behavior can be framed into one of our narratives, as narratives may have been either developed by ourselves from our own experiences or taken from other people [40, 46]. As regards certainties, they are assumptions that are spontaneously shown in whatever we say and do. Their primary feature consists in their being immune to doubt, for if someone called them into doubt, she could neither be certain of any judgment nor distinguish between true and false. Of course, doubts concerning certainties can be uttered, but not meaningfully. A legitimate doubt must be grounded; however, certainties are ungrounded because there are not grounds which are surer than the assertion of the certainty they are intended to justify. The characteristic sureness with which certainties are shown does not mean that they are grounded on reality, as we do not derive them from our experience through inductive reasoning [29]. Hence, certainty in Wittgenstein’s sense resembles what neuroscientists called “implicit memory”, for certainty constitutes “an attitudinal assurance that is either instinctual or automatic, and that should therefore be envisaged (…) in terms of reflex action” [51]. It should be borne in mind that certainties are wholly independent of mental states, as we constantly rely on countless certainties regardless of whether we think about them and even when we no longer wish that they make up our view of the world. After all, the mere idea of discovering that a certainty is wrong makes no sense within the corresponding world-picture. In other words, we can make a mistake regarding a grounded knowledge-statement, but not about a certainty. Thus, if someone said he had discovered his being dead, we would not regard such a statement as a mistake: instead, it would be an anomaly or a “grammatical gap” because then we could not know what he would still admit as evidence and what not [52]. Consequently, if someone held a doubt for which there is no room in our world-picture and admitted that something we said had removed his doubt, we would not know how or why.

This brief description of narratives and certainties suffices to realize that the latter are necessary for the existence of the former. Indeed, certainties also concern the meaning of words, as verbal communication would be impossible if a linguistic community called into doubt those meanings. Since narratives require the use of language, certainties are thus chronologically and logically prior to narratives. The phenomenological-hermeneutical interview is aimed at clarifying the structure of the interviewee’s life-world and thereby grasping the intended meaning of different events, associations and reactions [30, 53]. Yet this life-world cannot be developed if we do not already rely on a world-picture made up of the certainties assimilated by the individual until then. World-pictures entail *ontological* differences, as they do not only concern the meaning of words, but also establish what exists and with which characteristics. For instance, it stands fast for mentally healthy people that they are human beings, that they have heads and parents, and that they will die sooner or later. The nature of certainties is so basic that they cannot be assimilated through reflection: in fact, they are never acquired at will [54]. As a result of this, the individual only realizes the assimilation of a certainty once it has taken place, that is, when that certainty is already shown without any hint of doubt in whatever she does and says. Regarding individual narratives, they are usually not readily available texts, so that expert interviewers must coax them into being. Hence, narratives are very influenced by the interviewer’s questions not only where some party perceives there to be differences in status, but also because the interviewer may inadvertently provoke the inclusion and exclusion of topics, in addition to which many and important aspects of narratives can be misrepresented or lost in translation [55]. Furthermore, it should be noted that narratives can be distorted by the lens of time. Certainties, however, can be narrated or included in a narrative. It is true that certainties are enacted and can be uttered only for heuristic purposes, e.g. bringing up children or teaching a foreign language [56]. But as can be seen in the examples of the next paragraph, among those heuristic uses there could also be included some aspects of the patient’s world-picture that he himself may describe in the clinical interview in order to enrich his narrative.

The importance of taking certainties into account is particularly noticeable when the patient belongs to another culture and, therefore, shows some certainties that are different from ours. Thus, it has often been assumed that cross-cultural interaction should be managed by raising health care professionals’ cultural competence, [57, 58] but it is not clear what “cultural competence” means or how it should be taught, practiced or assessed [59, 60]. Regarding cross-cultural interaction within the context of end-of-life care, health care professionals often do not pay attention to specific ethno-cultural backgrounds, yet talk about such patients in homogenizing ways without knowing their ethno-cultural rituals in relation to dying, death and bereavement [61]. Of course, it is expected that communication and empathy with these groups will be simpler and more fruitful if health care professionals are acquainted with their certainties regarding the mentioned issues. Furthermore, there may emerge misunderstandings regarding the use of language that will escape detection unless enough attention is paid to the way in which people from another culture understand or interpret our words. It is therefore possible that some patients consider a cancer diagnosis as the confirmation that they will inevitably die in the short term, to the extent that they might regard an accurate explanation for this diagnosis as a hypocritical attempt to reassure them. If it is not certain for them that a cancer diagnosis amounts to the confirmation of an imminent death, they may end up admitting to be wrong; but if they are certain of such a thing, there will be no place in their world-picture for the possibility of making a mistake thereon, so that the practitioner’s explanation of the diagnosis will necessarily appear to them as a cynical deception or rather as a cruel mockery. In fact, these misunderstandings may engender inappropriate behaviors in patients: since they might become certain of their imminent death whatever they do, they may then be prone to leave aside healthy habits.

A further example can help appreciate the practical implications of taking into account intercultural differences in certainties. Let us suppose that a woman claims to be depressed because she concealed from her family that she has a job, and fears the estrangement from her culture as well as the drastic consequences of the fact that the family becomes aware of this. Nevertheless, this information alone is insufficient to clarify if this woman comes from a very traditional family such as those ultraconservative ones found in western culture, or from a community in which it is simply not conceived that its women devote themselves to any other task than household work. Despite appearances, both cases are very different because they concern respectively a quantitative and a qualitative matter. In the first case, the reference community could admit the possibility that its women work outside the home but would be very reluctant towards it, whereas in the second case the home community would regard such an idea as inconceivable, which entails that it automatically considers this woman as a completely alien person. It could be argued that, in the latter case, the intransigent community would have to admit the possibility of its female member working outside the home as soon as it noticed that this fact had taken place. The problem is that the community’s world-picture is blind to this possibility. Thus, the above-mentioned alienation would be so deep that the woman would have become for the members of her home community someone from whom they would not even know what to expect and with whom they would not know how to communicate. In addition, the woman would also have this alienated view of herself while she shared her home community’s world-picture, and it should not be forgotten that neither certainties nor world-pictures can be abandoned at will.

A similar example can be found in people belonging to religions with beliefs that clash with ours. As is widely known, Jehovah’s witnesses refuse blood transfusions. Yet this belief can be either grounded – so that the patient will have an argument related to some excerpts from the Gideon Bible – or already assimilated as an ungrounded certainty – in which case it will no longer be a mere argument but something so obvious as the fact that he is alive or what his name is. In the first case, it is an argument that the patient will have to defend with greater or lesser steadfastness because it cannot stand by itself, something that does not occur with certainties. In the second case, however, the patient will not feel the urge to utter the certainty, as he will take it for granted to such an extent that he will be unable to regard the intention to reject it as a wrong idea: instead, he will consider such intention as a totally *incomprehensible* aggression against his intimacy or his loved ones’. It is not surprising that this belief finally becomes a certainty, as health care professionals have often found that the religious faith of Jehovah’s witnesses is remarkably strong [62]. Thus, alternative modalities have been developed to treat the Jehovah’s witness patient with acute blood loss, [63] as empathic understanding must be used “to avoid acting against the patient’s will” [64]. Yet this understanding is also needed “to understand the patient’s illness or emotional reactions, (…) what is at stake for the patient (…) and to throw into relief the patient’s and the physician’s horizon”, [64] for which it is fundamental to discern whether a given belief constitutes a certainty. Indeed, this discernment would have been of outmost importance in encouraging empathy with patients in the last three examples.

## How to teach the management of certainties in second-order empathy

When medical schools design sessions and courses to enhance empathy in undergraduate medical students, it is highly recommended to start by clarifying that term. For this purpose, an interesting option is to emphasize the frequently overlooked difference between “empathy” and “sympathy” [65]. It is important to take a position on this issue, as there are authors whose definition of “empathy” coincides with the notion of “sympathy” presented above [66]. But, in my opinion, the reasons offered when I referred to sympathy justify that empathy is put into practice instead of it in clinical interviews. In fact, if certainties are taken into account, it is obvious that sympathy could not constitute a viable option because it would require sharing the patient’s certainties – which, as previously stated, cannot be done at will even if desired. Conversely, we can imagine what it would be like to share such certainties and get an idea of their consequences by requesting the necessary information from the patient. After defining the term “empathy”, it would be desirable to distinguish its dimensions, so that the proposal for enhancing empathy is as complete and balanced as possible. Four dimensions of empathy in the clinical context have been noted: the *emotional* (intrinsic ability to imagine the patients’ emotions, feelings and perspectives), the *moral* (motivation to want to empathize), the *cognitive* (correct identification and understanding of feelings), and the *behavioral* dimension (ability to convey understanding of those emotions and perspectives back to the patient) [43, 45, 67]. To illustrate how these four dimensions should be managed in clinical interview when trying to empathize through certainties, we could start with the moral dimension, which requires the health care professional to be motivated to empathize with the patient. Encouraged by this initial motivation, the physician could manage the cognitive dimension by attempting to identify the patient’s certainty regarding a specific issue. Once this information is made available, the practitioner may comply with the emotional dimension by imagining the patient’s emotions, thoughts and perspectives in a concrete situation bearing in mind the distinctive possibilities and impossibilities derived from her world-picture. Lastly, the behavioral dimension requires the physician to prove to the patient that he is aware of the emotions and perspectives generated by her certainties in a given context.

A fundamental problem when trying to empathize with patients is that doctors are too busy and have very little time for each consultation [68, 69]. However, 80% of patients do not need more than two minutes to develop a narrative if they can talk without interruptions [70]. Further problems have to do with the way in which practitioners listen to their patients. Thus, doctors interrupt patients when they have spoken for only twelve seconds on average [71]. Furthermore, physicians tend to rely on pre-conceptions to quickly classify patients and their complaints into sub-types [72]. It is therefore necessary to allow the patient to talk for longer and avoid the temptation to pigeonhole him. Stated otherwise, the practitioner should be attentive to the patient’s narrative, and subsequently ask specific questions to obtain the necessary information [34]. The issue to be clarified at this point is what information doctors should ask for in order to identify certainties. It would be misleading to carry out a direct assessment by asking a child questions such as “Is the Earth round?” or “What shape is the Earth?”, for the answers “Yes” or “Round” are not sufficient to distinguish whether she knows it – basing her knowledge on grounds – or it stands fast for her – because the Earth is already round in her world-picture [73]. Keeping this in mind, recourse should be made in the clinical setting to an indirect assessment that could be implemented in at least three ways:

- By focusing on the patient’s spontaneous reactions to the questioning of a certainty either in real cases or in cases raised along the interview. If he really has acquired that certainty, when it is called into doubt he should react with the same perplexity we would show if it were put into question that the Earth is round or what our name is.
- By asking what he would regard as the discovery of a mistake about a specific certainty. If it is not one of his certainties, he should know what mistakes thereon would be like, whereas he will be unable to conceive any mistake if it stands fast for him. In either case, we will understand in more detail the patient’s world-picture.
- By asking what happened in the past or what could happen in future when a common certainty had been infringed or an alleged one had been manifested. Information of these consequences will help us know the patient’s perspective – derived from his world-picture – on some facts and possibilities.

This kind of inquiries may allow the physician to enrich the patient’s narrative in order to understand him better. To get acquainted with such inquiries, we can use a number of resources whose effectiveness on the teaching of empathic skills in medical education has been proved. Thus, communication skill workshops emphasizing the behavioral dimension of empathy have been quite successful [65]. In this vein, role-modeling was the most effective way for medical students to acquire empathic skills by rehearsing what they had previously learned [74]. One of the reasons why empathy declines during medical training is precisely the lack of role models who exemplify the positive role of empathy in medical practice [21]. It would therefore be advisable that doctors with expertise in managing certainties participate in training sessions as models. Such models should also help medical students to understand that empathy will only be truly effective if it is perceived by the patient [65]. There are tools for measuring patients’ perceptions of medical empathy, [75] but they evidently do not take into account the management of certainties. Hence, feedback from expert testers regarding certainties should also be requested for training purposes. In fact, the evaluation of empathy can be enriched and supplemented through reflective practice, challenging cases, decision moments, and raters training to provide feedback [76].

It should not be forgotten that certainties cannot be modified at will, so that it would be a serious mistake to think that the patient must modify her certainties as the physician deems appropriate even before leaving his office. In fact, the respect which the patient deserves must prevent the doctor from being tempted to impose his community’s world-picture in a case of cross-cultural interaction or in the case of the Jehovah’s witnesses discussed above. But if the patient is willing to adopt a given certainty, such process could be facilitated by locating and showing resistances subtly – i.e. without disturbing nor, above all, without ridiculing her – through indirect assessment [77]. To this end, it would be illustrative to briefly indicate strengths, weaknesses and the main practical consequences of both world-pictures, focusing on the new certainty. A relevant testimony in favor of the target certainty may contribute to facilitate its acquisition in the foreseeable future, yet it is important that the testimony is tactful enough to avoid that the patient feels embarrassed by her wish to modify her world-picture.

## Conclusion

Over the last years narrative medicine has experienced substantial growth in the medical literature [49, 78]. After all, a growing number of doctors pay attention to their patients’ narratives to make a more accurate diagnosis and, by extension, to design an effective treatment plan [53]. As has been shown in this paper, certainties may play a role in narrative medicine by contributing to enhance second-order empathy. Nevertheless, the task of the practitioner would be absurd, not to say incomprehensible, if she took full account of the patient’s certainties but were not interested in his narratives: in such a case, the patient’s distinctive certainties would remain decontextualized and useless from a clinical point of view because they would lack a narrative in which to be embedded. Although each patient has his own and non-transferable narratives, not all patients will show certainties that are alien to the doctor’s world-picture and that are also necessary to understand their narrative. However, there are differences between world-pictures that can be very relevant to foster second-order empathy, so that the practitioner should give them due consideration. Last but not least, certainties constitute a dimension that supplements the definition of empathy, which is of the utmost importance because researchers have nearly unanimously noted that future research in this field would be bolstered if higher conceptual clarity were reached.
